# Hemostatic disturbances in traumatic brain injury: from mechanism to management

**DOI:** 10.1007/s00701-025-06549-w

**Published:** 2025-05-19

**Authors:** Alexander Fletcher-Sandersjöö, Jennifer Sebghati, Eric Peter Thelin

**Affiliations:** 1 Department of Clinical Neuroscience, Karolinska InstitutetBioclinicum J5:20, 171 64 Solna, Stockholm Sweden; 2 Department of Neurosurgery, Karolinska University Hospital, Stockholm, Sweden; 3 Medical Unit Neurology, Karolinska University Hospital, Stockholm, Sweden

**Keywords:** Traumatic brain injury, Coagulopathy, Hemostasis, Contusion, Bleeding, Hyperfibrinolysis

## Abstract

Coagulopathy following traumatic brain injury (TBI) is increasingly being recognized as a determinant of hematoma expansion and outcome. Unlike systemic trauma, where coagulopathy is often driven by hemorrhagic shock, TBI appears to induce a unique brain-derived hemostatic response. In this review, we discuss the mechanisms underlying TBI-induced coagulopathy, its diagnostic challenges, and association with hematoma expansion. We further evaluate evidence from randomized trials targeting coagulopathy in TBI, including interventions such as tranexamic acid, plasma, recombinant Factor VIIa, and fibrinogen. While several studies show proof of concept, clinical benefit remains inconsistent, likely due to issues of timing, heterogeneity, and underpowered study design. Moving forward, ultra-early and individualized approaches guided by real-time hemostatic monitoring may offer the most promising path. A better understanding of the temporal and mechanistic dynamics of coagulopathy will be essential for improving treatment strategies and patient outcomes.

**Supplementary Information:**

The online version contains supplementary material available at 10.1007/s00701-025-06549-w.

## Introduction

Coagulopathy in traumatic brain injury (TBI), broadly defined as any disruption in normal hemostatic function, has gained increasing attention due to its association with both hematoma expansion [77] and poor outcome [30, 44]. It has been the target of several recent randomized controlled trials (RCTs) [11, 48, 56], but with no consistent clinical benefit. Identifying which patients are at the highest risk for hematoma expansion, and determining when and how to intervene, remain key clinical challenges. This narrative review examines our current understanding of TBI-induced coagulopathy. It begins with an overview of normal hemostasis, and then explores how TBI disrupts coagulation dynamics, its role in hematoma expansion, diagnostic strategies, and current therapeutic approaches. Although a large proportion of TBI patients, particularly elderly individuals, present with anticoagulant- or antiplatelet-associated coagulopathy, the current review focuses on trauma-induced disturbances, which have distinct pathophysiological features and therapeutic implications.

## Hemostasis in brief: core concepts relevant to TBI

### Normal hemostasis

Hemostasis is a finely tuned process that ensures effective clot formation while preventing pathological thrombosis [12]. It consists of three overlapping stages: primary hemostasis, secondary hemostasis, and fibrinolysis. In primary hemostasis, platelets adhere to the site of vascular injury through binding of their glycoprotein Ib (GPIb) receptor to von Willebrand factor (vWF), which is itself anchored to exposed subendothelial collagen. This triggers platelet activation, leading to the expression of glycoprotein IIb/IIIa (GPIIb/IIIa) receptors, which facilitate platelet-to-platelet aggregation through fibrinogen bridging. Activated platelets also release granules containing substances that amplify activation of nearby platelets independent of vWF, thereby initiating a self-sustaining cascade. Platelet aggregation through GPIIb/IIIa ultimately forms the platelet plug. While this effectively seals minor endothelial disruptions, more extensive injuries require reinforcement by fibrin [12, 64].

The fibrin reinforcement comes from secondary hemostasis, traditionally conceptualized by the coagulation cascade. While once divided into intrinsic and extrinsic pathways that merge at a common endpoint, the contemporary model emphasizes three overlapping phases. In the initiation phase, tissue factor (TF) exposed at the site of vascular injury binds to factor VIIa, which activate factors IX and X. Factor Xa then forms the prothrombinase complex with factor Va, converting prothrombin to thrombin. During the amplification phase, the generated thrombin further activates platelets and cofactors, particularly factors V and VIII, resulting in the production of large amounts of Factor Xa. In the propagation phase, the produced prothrombinase complex generates large amounts of thrombin, which converts fibrinogen to fibrin, reinforcing the platelet plug and forming a stable clot [8, 12].

Once the clot has fulfilled its role, fibrinolysis ensures that it is removed to restore normal blood flow. Plasmin, generated from plasminogen by tissue plasminogen activator (tPA), is the key enzyme responsible for degrading fibrin. This process is tightly regulated: plasminogen activator inhibitor-1 (PAI-1) inhibits tPA, while alpha-2-antiplasmin neutralizes plasmin. Together, these inhibitors prevent premature or excessive fibrinolysis. In healthy individuals, a delicate balance between clot formation and dissolution is maintained – sufficient to stop bleeding without promoting unnecessary thrombosis [12, 28].

### Monitoring hemostasis in TBI

Supplementary Table 1 presents a comprehensive, though not exhaustive, overview of tests and biomarkers relevant to hemostasis in TBI. Some are part of routine clinical practice, while others are only used in research.

In the clinical setting, hemostasis is typically first assessed using prothrombin time (PT)/international normalized ratio (INR), activated partial thromboplastin time (APTT), platelet count, and fibrinogen [5, 13, 38]. These offer a general view of coagulation factor levels and platelet availability. However, while PT/INR and APTT can indicate factor deficiencies or inhibition, they do not fully reflect the kinetics of clot formation or platelet function. For instance, a normal PT/INR does not rule out the presence of coagulopathy [52].

To gain more detailed insights, markers such as thrombin–antithrombin (TAT), prothrombin fragment 1 + 2 (F1 + 2), and tPA can be measured. Elevated TAT or F1 + 2 suggest heightened thrombin generation, while increased tPA levels point to active fibrinolysis [5, 38]. Specialized assays are also available for assessing platelet function and global hemostatic capacity, including viscoelastic tests such as thromboelastography (TEG) and rotational thromboelastometry (ROTEM), which monitor clot development, strength, and breakdown in real time [5, 38]. These tools can detect conditions like hyperfibrinolysis or platelet dysfunction that may not be apparent through standard coagulation tests. In research or selected clinical scenarios, further granularity can be obtained by measuring regulators of fibrinolysis such as α₂-plasmin inhibitor (α₂-PI) or the plasmin–α₂-PI complex (PIC), as well as natural anticoagulants such as antithrombin, protein C, and protein S.

## Key features of TBI-induced coagulopathy

As early as 1974, Goodnight and colleagues reported reductions in fibrinogen, factor V, and factor VIII in TBI-patients [24]. Subsequent studies have confirmed a transient elevation in APTT and PT [29, 40, 47], increased levels of TAT and F1 + 2 [23, 25, 49, 59, 63], and reduced fibrinogen concentrations [42, 47] during the first hours after injury [18]. These findings suggest a disruption of the coagulation cascade, likely due to excessive thrombin generation – a process known as consumptive coagulopathy. At the same time, an exaggerated fibrinolytic response appears, with studies reporting elevated levels of D-dimer [17, 23, 34, 47, 49, 66, 70], PAI-1, and t-PA [6, 17, 23, 34] within the same time period [18]. In addition, both platelet count [18] and function [9, 37, 39, 71] have been shown to decline over time, although these changes are typically more gradual and less pronounced. Thus, evidence points to three interwoven reactions that can predispose TBI-patients to bleeding: consumptive coagulopathy, hyperfibrinolysis and platelet dysfunction. The dynamic nature of these disturbances is further underscored by findings that the proportion of patients with coagulopathy can double within 24 h of injury [27]. This suggests that while some cases present with coagulopathy on admission, others develop it as the injury evolves, reflecting delayed or progressive hemostatic dysregulation.

It should also be noted that TBI has been associated with an increased risk of venous thromboembolism [19, 62] and ischemic stroke [3, 19, 35]. This shift from a hypocoagulable to a hypercoagulable state has been observed in both clinical studies and experimental models. Factors contributing to this transition likely include endothelial activation, platelet hyperreactivity, and fibrinolysis shutdown. The timing of this transition remains poorly defined, complicating efforts to balance the risks of hematoma expansion and thrombotic complications [44].

## Mechanism behand TBI-induced coagulopathy

Understanding the mechanisms that drive coagulopathy in TBI is essential for developing targeted interventions. While systemic mechanisms such as endothelial glycocalyx shedding, protein C activation, and complement–coagulation crosstalk have been proposed [44], these are likely more relevant to polytrauma with hemorrhagic shock or massive transfusion. In isolated TBI, coagulopathy appears to stem from brain-specific processes, including TF release and locally mediated hyperfibrinolysis. This section explores these mechanisms in greater detail.

TF is abundantly expressed in brain parenchyma, but normally sequestered behind the blood–brain barrier (BBB) [15]. Following TBI, disruption of the BBB is believed to allow TF to enter the circulation, where it binds to factor VII to form the TF–FVIIa complex. This complex catalyzes the activation of factor X to Xa, leading to the assembly of the prothrombinase complex, which, together with factor Va, converts prothrombin into thrombin, thereby initiating the coagulation cascade [8, 12] and, if overactivated, potentially leading to consumptive coagulopathy. This hypothesis has gained support from several experimental studies. Hubbard et al., showed that thrombin generation following TBI was dependent on TF carried by extracellular vesicles, where peak levels occurred between 6 and 24 h post-injury depending on severity [33]. Tian et al. further demonstrated that brain-derived microparticles (BDMPs) expressing both TF and phosphatidylserine are released into the bloodstream after TBI, which induce consumptive coagulopathy when injected in uninjured mice [69]. Zhou et al. expanded on this mechanism, showing that pharmacologic clearance of BDMPs mitigated coagulopathy and improved survival in experimental TBI models [79]. Human studies support the translational relevance of these findings. Two investigations by Nekludov et al. reported elevated levels of BDMPs, along with TF-bearing microparticles of endothelial and platelet origin, in both systemic and cerebrovenous blood of patients with isolated TBI [50, 51].

While the above studies focus on the coagulation cascade, others have highlighted disturbances in anticoagulant and fibrinolytic balance. A prospective study by Albert et al. found that TBI patients who developed coagulopathy exhibit enhanced thrombin generation, depletion of endogenous anticoagulants such as protein S, and increased fibrinolytic activity, including elevated tPA levels and reduced plasmin inhibition, suggesting a state of insufficient regulation rather than isolated activation [2]. A murine study by Hijazi et al. further suggested that hyperfibrinolysis may be directly triggered by the release of the plasminogen activators tPA and uPA from the injured brain, rather than occurring secondarily to clot formation. Mice lacking these enzymes were protected from hematoma progression, and treatment with a catalytically inactive tPA variant reduced fibrinolysis and improved outcomes [32].

## Clinical risk factors for TBI-induced coagulopathy

The development of coagulopathy in TBI is influenced by both patient-specific characteristics and injury-related factors, and is likely a product of multiple interacting mechanisms. Among the most consistently identified risk factors is injury severity: lower Glasgow Coma Scale (GCS) scores [10, 61, 67, 72], higher head Abbreviated Injury Scale (AIS) [72], and the presence of abnormal pupils [16] have all been independently associated with an increased likelihood of coagulopathy. Age also plays a key role. Older patients appear more prone to developing coagulopathy after TBI [16, 72], a finding of particular relevance given the demographic shift where TBI is now increasingly more common in the elderly [54].

Radiographic injury patterns can also provide insight. Patients with higher Marshall CT Classification [61], traumatic subarachnoid hemorrhage, brain edema, and significant midline shift [67] are more prone to coagulopathy. Moreover, penetrating TBI has been linked to a significantly higher incidence than blunt trauma [40]. Finally, the systemic response to trauma can further disrupt hemostasis. Hypotension [16, 67, 72], hypothermia [44], high-volume fluid resuscitation [72], metabolic acidosis [41], and hyperglycemia [4, 10] have all been associated with increased coagulopathy risk.

## Association between hematoma expansion and TBI-induced coagulopathy

Identifying which aspects of coagulopathy influence hematoma expansion will be key to targeted treatment. Both consumptive coagulopathy and hyperfibrinolysis appear to manifest within the first few hours after injury [18], i.e. when the risk of hematoma expansion is highest [20]. Supporting this, studies have identified correlations between hematoma expansion and abnormalities in PT, APTT [7, 36], Factor VII activity [74], and fibrinogen [68]. Elevated D-dimer levels have also been linked to hematoma expansion in several studies [17, 68, 75].

In contrast, an association between TBI-induced platelet dysfunction and hematoma expansion has not yet been confirmed [39]. This may be due to platelet dysfunction occurring later on when most hematomas have stabilized [18, 20], or because levels don’t fall low enough to functionally impair primary hemostasis. Interpreting platelet function tests is further complicated by the fact that the assays were developed to assess antiplatelet therapy, complicating the establishment of clinically relevant cutoffs for TBI. Highlighting this, the recently updated edition of the European guideline on the management of major bleeding and coagulopathy following traumatic injury recommend that the routine use of point-of-care platelet function devices be avoided [55].

Scoring models aimed at predicting contusion expansion offer additional insight into the clinical relevance of coagulopathy in TBI, although the findings on which markers are most relevant remain inconsistent. Wei et al. identified D-dimer (> 5 mg/L) as the sole coagulation-related predictor of contusion growth, while platelet count, PT/INR, APTT, thrombin time, and fibrinogen were not [73]. He et al. reported PT/INR as a significant predictor but found no association with APTT or platelet count [31]. A model by Zhang et al. supported the predictive value of PT/INR and fibrinogen [78], whereas Yuan et al. included D-dimer, prolonged PT/INR, and reduced platelet count in their model [75]. Lastly, two studies have explored a broader coagulopathy definition rather than single markers. Zhu et al. found that patients meeting one or more criteria (APTT > 1.5 × normal, PT > 1.5 × normal, platelet count reduced by 25%, or fibrinogen < 1.0 g/L) were more likely to experience contusion expansion [80]. However, Sheng et al., using slightly different thresholds (INR > 1.2, APTT > 36 s, or platelet count < 120 × 10⁹/L), found no significant association [60].

## Management strategies for TBI-induced coagulopathy

There is currently no consensus on how best to manage coagulopathy in TBI. The evidence base remains limited, and treatment strategies likely vary widely between centers and clinicians. Management often depends on factors such as the severity and pattern of coagulopathy, the presence of active bleeding, and the availability of real-time coagulation monitoring. The following section summarizes the limited RCT data currently available.

### Tranexamic acid

The potential for tranexamic acid (TXA) to reduce hyperfibrinolysis and hematoma expansion in TBI has been the subject of extensive investigation. By inhibiting plasmin-mediated fibrin degradation, TXA could theoretically stabilize clot formation and limit hematoma growth. Following the CRASH-2 trial, which demonstrated a survival benefit with TXA in polytrauma patients with significant extracranial bleeding [53], there was optimism that similar benefits could be observed in isolated TBI. However, trials have yielded mixed results. CRASH-3 remains the largest RCT of TXA in TBI, enrolling over 12 000 patients and using a 1 g loading dose followed by a 1 g infusion over 8 h. While TXA did not reduce head-injury-related mortality overall, a subgroup analysis suggested a survival benefit in mild-to-moderate TBI [11], despite no observed effect on hematoma expansion [45]. However, interpretation of CRASH-3 is complicated by several methodological issues, including a mid-trial change in inclusion criteria, narrowing the treatment window from 8 to 3 h, and reliance on disease-specific mortality.

The TBI-EPIC trial further explored prehospital TXA in moderate-to-severe TBI, randomizing patients into three groups: a 1 g bolus + 1 g infusion (mirroring CRASH-3), 2 g bolus, or placebo. The study found no significant difference in 6-month functional outcome [56], overall mortality [56], or hematoma expansion [57]. Moreover, TXA did not appear to influence fibrinolysis markers [14]. However, a subgroup analysis of patients with CT-confirmed intracranial hemorrhage found a lower mortality rate in the 2-g bolus group (17%) compared to the 1 g + 1 g group (26%) and placebo (27%) [57]. These findings have informed recent updates to the UK National Institute for Health and Care Excellence (NICE) guidelines, which now recommend 2-g intravenous TXA bolus within 2 h of head injury (prior to imaging) in adults with suspected moderate or severe TBI. The recommendation acknowledges the uncertainty in the evidence base but reflects a pragmatic approach in the absence of clear harms [1].

Importantly, no increase in thrombotic complications has been reported in the largest TXA trials in TBI. Neither CRASH-3 nor TBI-EPIC observed a higher rate of venous thromboembolism or other vascular occlusive events in the TXA group compared to placebo [11, 56]. These findings are supported by a large systematic review and meta-analysis of 234 randomized trials involving over 100,000 bleeding patients, which found no increased risk of thrombotic events with TXA (RR 1.00, 95% CI 0.93–1.08), including no significant dose-dependent effect in meta-regression analysis. In subgroup analyses of trauma and TBI, the relative risk for thrombotic events was similarly neutral (RR 0.89, 95% CI 0.69–1.17), suggesting that TXA is unlikely to increase thrombotic risk in this population, even at higher doses [46].

### Prehospital thawed plasma

The Prehospital Air Medical Plasma (PAMPer) trial evaluated the use of thawed plasma during air medical transport in trauma patients at risk of hemorrhagic shock. Compared to standard crystalloid-based resuscitation, prehospital plasma was associated with a significant reduction in 30-day mortality [65]. A prespecified subgroup analysis also showed that plasma was associated with improved survival in the TBI-cohort [26]. However, the greatest benefit was seen in patients with both TBI and concomitant extracranial injuries, making it difficult to determine whether the observed effect was due to correction of TBI-specific coagulopathy or systemic resuscitative support in polytrauma. Notably, plasma administration improved INR values but did not significantly affect TEG markers, raising questions about its impact on coagulopathy. Importantly, the trial did not report data on hematoma expansion – an effect that, if demonstrated, would have strengthened the rationale for its use.

### Coagulation factor concentrate

Beyond plasma, the consumptive coagulopathy observed in TBI suggests that targeted clotting factor replacement could help reduce hematoma expansion. An early trial of recombinant Factor VIIa (rFVIIa) enrolled 97 patients with contusions ≥ 2 mL and randomized them to rFVIIa or placebo across five escalating dose tiers (40–200 µg/kg). While no statistically significant differences were found in mortality, adverse events, or functional outcomes, there was a trend toward reduced absolute contusion expansion in the rFVIIa group (10.1 mL vs. 21.0 mL) [48]. However, a higher incidence of deep vein thrombosis (8% vs. 3%) raised concerns about thrombotic risk, limiting enthusiasm for its clinical use. Nonetheless, the trial provided early evidence that hemostatic intervention may reduce hematoma expansion.

A more recent study examined low-dose rFVIIa (20 µg/kg) in 87 patients with isolated moderate-to-severe TBI and laboratory-defined coagulopathy (INR > 1.2, APTT > 36 s, or platelet count < 100 × 10⁹/L). Hematoma expansion occurred in 18% of treated patients versus 39% in the control group, suggesting a possible benefit. However, the study’s non-randomized, non-blinded design introduces the risk of substantial bias. Additionally, thromboembolic events were only monitored for 72 h, potentially missing delayed complications, and baseline comparability between groups was unclear [76]. Taken together, these findings underscore the need for a larger RCT of low-dose rFVIIa in TBI.

### Fibrinogen

Hypofibrinogenemia has been associated with hematoma expansion in TBI, making fibrinogen replacement a potential therapeutic strategy. To date, only one study has directly evaluated this approach. In a randomized trial of 71 patients with severe isolated TBI and fibrinogen levels < 200 mg/dL, fibrinogen administration was compared to standard care. Hematoma expansion, defined as either > 5% volume increase of existing intracranial lesions or gross transformation of small hemorrhages into larger ones, was significantly less common in the fibrinogen group (22.2% vs. 65.7%) [58]. However, the lack of blinding introduces a risk of bias, as knowledge of group assignment may have influenced management. Additionally, thromboembolic events were not monitored beyond clinical suspicion, leaving uncertainty regarding potential thrombotic complications such as stroke or deep vein thrombosis.

## Points of perspective

The past decades have brought significant advances in our understanding of TBI-induced coagulopathy, but translating these insights into treatment remains a challenge. This is partly due to the complex nature of post-injury hemostatic disturbances, and partly due to the limitations of existing diagnostics and therapies.

Despite strong pathophysiological rationale, trials of TXA have failed to demonstrate a clear benefit in limiting hyperfibrinolysis and hematoma expansion. One possibility is that TXA alone is insufficient to counteract the multifaceted hemostatic derangements seen in TBI, which include not only hyperfibrinolysis, but also consumptive coagulopathy and platelet dysfunction. Pharmacodynamic effect may be another key. A 2-g bolus seemed to provide the best effect in the TBI-EPIC trial [57], raising the possibility that higher single-dose regimens may be more effective than prolonged low dose infusions in suppressing early, brain-driven hyperfibrinolysis. Given the intensity and speed of tPA release following TBI [32], even higher doses, such as 3–4 g TXA, might be needed to achieve sufficient plasma concentrations to meaningfully blunt the fibrinolytic surge. This warrants further investigation in dose-ranging studies. The trials of rFVIIa [48] and fibrinogen concentrate [58] provide further proof of concept that targeting coagulopathy can limit hematoma expansion in TBI. However, both studies were limited by sample size. Future trials should also be consistent in how they define and measure hematoma expansion, as we have found that methodological variability in volume estimation and differences in using absolute versus relative expansion may influence findings [21, 22]. Moreover, while this review focuses on isolated TBI, it remains unclear how emerging strategies such as whole blood transfusion in polytrauma settings may influence brain-specific coagulopathy. This represents an important area for future research.

Going forward, a key question will be how to time and individualize hemostatic interventions. The window for hematoma expansion is often short, typically only a few hours [20], yet few trials have intervened at this early stage. Studies will have to prioritize ultra-early enrollment, potentially in the prehospital setting, to capture this window. At the same time, not all patients develop coagulopathy. A test-then-treat strategy, guided by viscoelastic testing or other rapid diagnostics, may offer a more balanced approach – limiting overtreatment while still enabling early action. Indeed, a recent meta-analysis showed a 20% reduction in mortality with viscoelastic testing-guided bleeding management in polytrauma [43]. However, while point-of-care coagulation testing holds promise, its widespread implementation remains unrealistic in many settings, as the majority of TBI cases occur in low-resource environments where this is neither logistically feasible nor a healthcare priority. The cost of treatment is also a key consideration in these settings, where low-cost therapies such as TXA may be more feasible than expensive agents like factor concentrates, despite potential superior efficacy. Future research must balance scientific advancements with pragmatic solutions that can be effectively integrated into diverse healthcare systems, ensuring that progress in hemostatic management benefits the full spectrum of TBI patients.

## Supplementary Information

Below is the link to the electronic supplementary material.

Supplementary file1 (DOCX 330 KB)

## Declarations

### Data availability

No datasets were generated or analyzed during the current study.

## Abbreviations

*AIS*: Abbreviated Injury Scale
*APTT*: Activated Partial Thromboplastin Time
*BBB*: Blood–Brain Barrier
*BDMPs*: Brain-Derived Microparticles
*CAT*: Calibrated Automated Thrombogram
*CT*: Computed Tomography
*DAI*: Diffuse Axonal Injury
*F1 + 2*: Prothrombin Fragment 1 + 2
*GCS*: Glasgow Coma Scale
*INR*: International Normalized Ratio
*OHP*: Overall Hemostatic Potential
*PAI-1*: Plasminogen Activator Inhibitor-1
*PAP*: Plasmin–Antiplasmin Complex
*PIC*: Plasmin–α₂-Plasmin Inhibitor Complex
*PT*: Prothrombin Time
*rFVIIa*: Recombinant Factor VIIa
*ROTEM*: Rotational Thromboelastometry
*TAT*: Thrombin–Antithrombin Complex
*TBI*: Traumatic Brain Injury
*TEG*: Thromboelastography
*TF*: Tissue Factor
*TF–FVIIa*: Tissue Factor–Factor VIIa Complex
*tPA*: Tissue Plasminogen Activator
*TXA*: Tranexamic Acid
*uPA*: Urokinase Plasminogen Activator
*vWF*: Von Willebrand Factor
