# Supplementary material for: Hemostatic disturbances in traumatic brain injury: from mechanism to management
Source: Acta Neurochir (Wien). 2025 May 19;167(1):146. doi: 10.1007/s00701-025-06549-w (PMC12089152; doi:10.1007/s00701-025-06549-w)
Supplement: Supplementary file 1 — Supplementary file1 (DOCX 330 KB) [file 701_2025_6549_MOESM1_ESM.docx]

**Supplementary table 1: Measurements of hemostasis used in studies of TBI-induced coagulopathy**

| **Test name** | **Aspect of hemostasis measured** |
| --- | --- |
| Platelet count | Quantitative component of primary hemostasis. |
| Platelet function tests | Qualitative performance of platelets. Examples include light transmission aggregometry, Multiplate, and VerifyNow. |
| APTT (Activated Partial Thromboplastin Time) | Assesses factors in the intrinsic and common pathways of secondary hemostasis. Prolonged values may indicate factor deficiency or inhibition. |
| PT/INR (Prothrombin Time/International Normalized Ratio) | Primarily reflects the extrinsic and common coagulation pathways. |
| Fibrinogen | Key substrate in secondary hemostasis. Low levels suggest consumption or hyperfibrinolysis, whereas high levels can be an acute-phase response. |
| Antithrombin | Natural inhibitor of thrombin and Factor Xa. Low levels can reflect consumption or genetic deficiency. |
| Factor activity assays | Specific measures of individual coagulation factors. Reduced activity suggests deficiency or consumption. |
| Protein C and Protein S | Vitamin K-dependent natural anticoagulants. Deficiencies or functional impairments can shift toward a prothrombotic state. |
| Plasminogen | Proenzyme converted to plasmin, the main fibrinolytic enzyme. Low levels can limit fibrinolysis, whereas high consumption may indicate hyperfibrinolysis. |
| D-dimer | Fibrin degradation product reflecting ongoing fibrinolysis. Elevated in many conditions and requires clinical correlation. Particularly high levels may suggest hyperfibrinolysis or extensive clot turnover. |
| Fibrin monomers | Soluble intermediates formed before cross-linked fibrin. Elevated levels can indicate ongoing coagulation with insufficient clearance, sometimes preceding overt coagulopathy. |
| TAFI (Thrombin Activatable Fibrinolysis Inhibitor) | Reduces plasminogen binding to fibrin, inhibiting fibrinolysis. |
| PAI-1 (Plasminogen Activator Inhibitor-1) | Inhibits tPA and uPA, thus reducing fibrinolysis. Elevated levels may be linked to thrombosis; low levels can facilitate excess fibrinolysis. |
| α₂-Plasmin Inhibitor (α₂-PI) | Principal physiological inhibitor of plasmin. Low α₂-PI permits hyperfibrinolysis; elevated α₂-PI can excessively curb fibrin breakdown. |
| Plasmin–α₂-PI Complex (PIC) | Complex formed when plasmin is neutralized by α₂-PI. Elevated levels indicate robust plasmin activity followed by inhibition, reflecting active fibrinolysis. |
| PAP (Plasmin–Antiplasmin Complex) | Another measure of plasmin bound to its inhibitor, antiplasmin. Similar to PIC in indicating ongoing fibrinolysis. |
| TAT (Thrombin–Antithrombin Complex) | Forms when thrombin is neutralized by antithrombin. Elevated levels suggest high thrombin generation, reflecting an activated coagulation cascade. |
| F1 + 2 (Prothrombin Fragment 1+2) | Released when prothrombin is cleaved to thrombin, indicating ongoing thrombin generation (secondary hemostasis). |
| Thrombin Generation Test (TGT) | Also called Calibrated Automated Thrombogram (CAT). Evaluates the time course and magnitude of thrombin production in plasma. Largely a research tool, but offers insights into overall coagulant capacity. |
| tPA and uPA (tissue-type and urokinase-type plasminogen activator) | Activators that convert plasminogen to plasmin, thereby driving fibrinolysis. Elevated levels indicate hyperfibrinolysis. |
| TEG / ROTEM | Viscoelastic assays that provide a global, real-time assessment of clot formation, strength, and lysis in whole blood |
| Overall Hemostatic Potential (OHP) | A plasma-based assay that quantifies the net balance between fibrin formation and fibrinolysis. Although referenced in TBI research, it is not broadly available. |
